# Case Report: Pathogenic *MYH9* c.5797delC Mutation in a Patient With Apparent Thrombocytopenia and Nephropathy

**DOI:** 10.3389/fgene.2021.705832

**Published:** 2021-07-28

**Authors:** Pingping Ren, Hongjun Chen, Yucheng Wang, Cuili Wang, Shi Feng, Hong Jiang, Jianghua Chen

**Affiliations:** ^1^Kidney Disease Center, the First Affiliated Hospital, College of Medicine, Zhejiang University, Hangzhou, China; ^2^Key Laboratory of Nephropathy, Hangzhou, China; ^3^Institute of Nephropathy, Zhejiang University, Hangzhou, China

**Keywords:** MYH9-related disease, exon mutation, nephropathy, thrombocytopenia, autosomal dominant disease

## Abstract

MYH9-related disease or disorder (MYH9-RD) is an autosomal dominant disease caused by mutations in the *MYH9* gene. Mutations in this gene initially affect the hemic system, and other manifestations may evolve with age. Here, we report the case of a 46-year-old Chinese woman with MYH9-RD who was primarily misdiagnosed with idiopathic thrombocytopenia purpura. Exome sequencing of the patient, and the mother and son of the patient revealed a deletion mutation c.5797delC (p. R1933Efs^*^15) in exon 41 (encoding non-helical tailpiece, NHT) of the *MYH9* gene, which consequently led to a frameshift mutation. To the best of our knowledge, this mutation has been reported in Italy once, while the substitution mutation c.5797 C>T is the most frequent mutation. Mutations that affect the NHT region cause thrombocytopenia throughout life; however, our patient presented with a more severe phenotype than previously reported, including thrombocytopenia, inclusion bodies in neutrophils, sensorineural hearing loss, nephropathy, and abnormal liver enzymes. Our goal in the current case is to prevent further progression of renal involvement and to identify other affected members in this family to provide early intervention. This case may raise awareness of MYH9-RD when diagnosing thrombocytopenia and improve our understanding of this condition.

## Introduction

The human *MYH9* gene is located on chromosome 22q12.3 and encodes non-muscle myosin heavy chain IIA, which is widely expressed in more than 27 different tissues. As a cytoskeletal contractile protein, it plays an essential role in cell adhesion, cell migration, and tissue architecture (Vicente-Manzanares et al., 2009). Each heavy chain comprises two domains: the N-terminal head domain (HD), which consists of a global motor domain and a neck domain, and a C-terminal tail domain (TD), including a long α-helical coiled-coil region ending with a non-helical tailpiece (NHT) (Pecci et al., 2018).

MYH9-related disease or disorder (MYH9-RD) is a group of diseases including May–Hegglin anomaly; Fechtner, Sebastian, and Epstein syndromes; and DFNA17, which are all caused by mutations in *MYH9* (Lalwani et al., 2000; Seri et al., 2000; Arrondel et al., 2002). However, these five named phenotypes cannot cover all possible manifestations as the phenotype of a person with an MYH9 pathogenic variant often evolves over time; hence, the term MYH9-RD has been proposed (Savoia and Pecci, 1993). The clinical picture of MYH9-RD is characterized by hematologic features consisting of platelet macrocytosis, thrombocytopenia, and inclusion bodies in neutrophil granulocytes, which are present at birth in all affected individuals, while some patients develop one or more extrahematological manifestations, including sensorineural deafness, cataract, and nephropathy, which eventually lead to end-stage renal disease (ESRD).

According to the Global Variome database, the MYH9 gene homepage (Global Variome shared LOVD), 149 mutants have been reported worldwide. Although the relationship between genotype and phenotype is not consistent, genotype–phenotype correlations are recognized to some degree, and an analysis based on a large case series of patients with MYH9-RD demonstrated that patients with mutations in the HD had a more severe phenotype and worse prognosis than those with the TD alterations. Among the mutation hotspots, position 1,933 at the NHT was associated with the lowest risk of developing nephritis or other non-congenital complications; thus, thrombocytopenia may remain the only manifestation in patients harboring this mutation (Pecci et al., 2008b, 2014). Excepting an R702 substitution is the only determinant of ESRD development; additional genetic or environmental factors are required for progression to ESRD in patients with coiled-coil substitutions (Pecci et al., 2014). Here, we present a case of an exon mutation c.5797delC in the NHT region of the *MYH9* gene in a patient with symptomatic thrombocytopenia, progressive kidney involvement, and other impaired organs. To the best of our knowledge, this mutation has been reported once.

## Case Presentation

### Clinical History and Laboratory Data

The patient was a 46-year-old Chinese woman. In November 2019, she was admitted to the department of hematology at our hospital due to thrombocytopenia, menorrhagia, and anemia. She was diagnosed with idiopathic thrombocytopenia purpura (ITP) and suspected inherited thrombocytopenia. Laboratory tests revealed a serum creatinine level of 158 μmol/L and trace urine protein without hematuria. As the main symptom was thrombocytopenia, and other manifestations were not recognized, she was treated for ITP. She received dexamethasone for 6 months, but her condition did not improve.

In May 2020, she received induced menopause treatment, and elevated serum creatinine and urine protein levels were found with no detailed information.

In June 2020, she was hospitalized again for further treatment at the local hospital; at this time, her serum creatinine level was 224 μmol/L, and urine protein was detected as “2+” with a quantitative test of urine protein indicating 2.4 g/day. Routine blood tests showed a significant decrease in the blood platelet count, which was 19 × 10^9^/L (normal range, 100–300 × 10^9^/L), and her hemoglobin level was indicative of mild anemia. The results of pure tone audiometry indicated mild hearing loss and moderate hearing loss in the right and left ears, respectively. Considering the above results, MYH9-RD was suspected.

**Figure 1:**
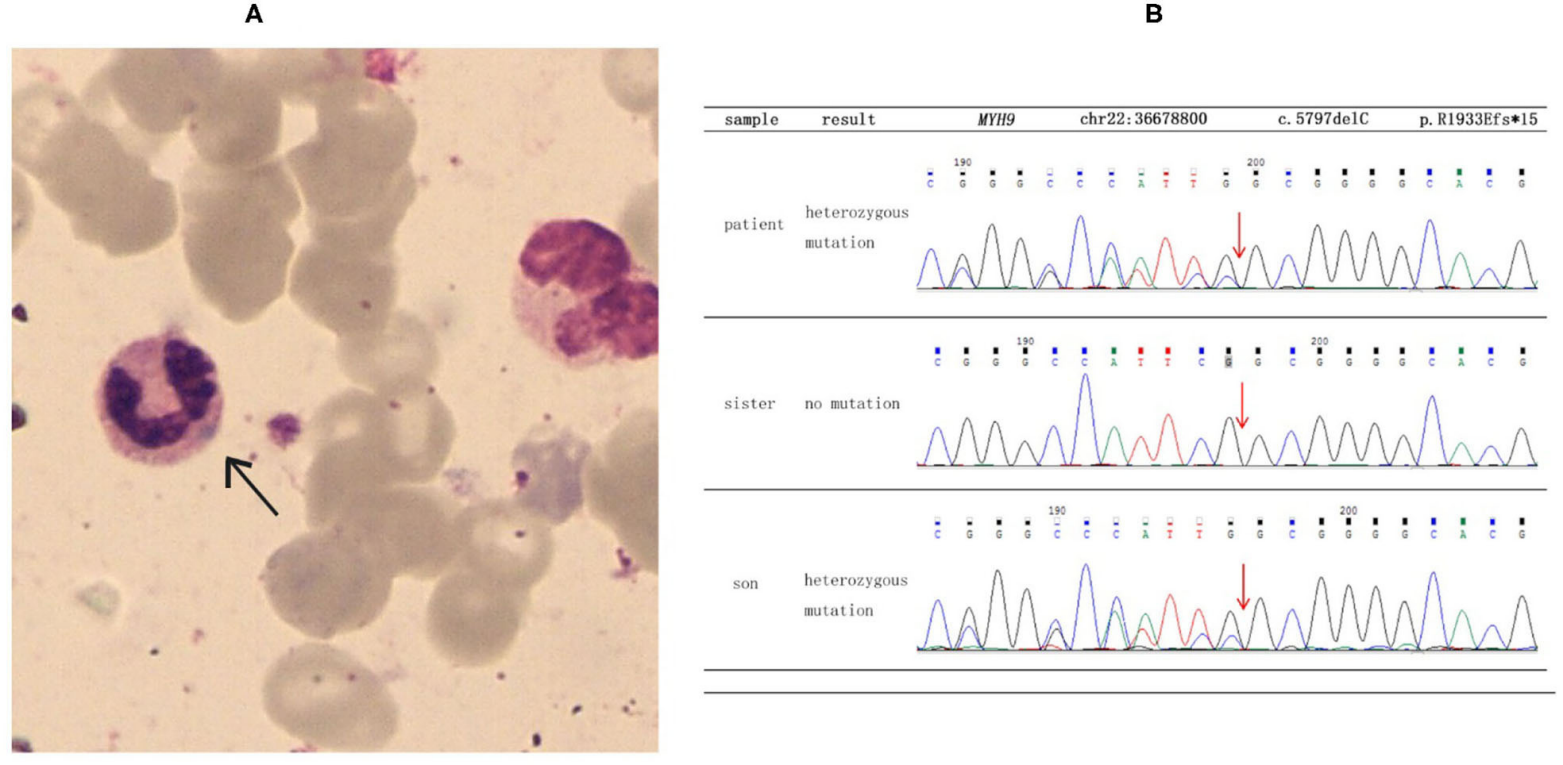
Results of blood smear and direct sequencing. **(A)** The black arrow indicates inclusion bodies in the neutrophils on the blood smear (Wright–Giemsa staining). **(B)** The results of direct sequencing of *MYH9* exon 41 in the proband and other family members. The proband and her son were both heterozygous for a deletion mutation of c.5797delC, which then led to a frameshift mutation and caused a prematurely truncated protein p. R1933Efs^*^15.

She was referred to the nephrology department for further diagnosis and therapy. Detailed examination revealed similar results as above: a platelet count of 27 × 10^9^/L, urine protein level of “3+,” and serum creatinine level of 265 μmol/L [estimated glomerular filtration rate (GFR), 18 ml/min). Another laboratory finding was elevated liver enzymes with an alanine aminotransferase (ALT) level of 78 U/L and aspartate aminotransferase (AST) level of 62 U/L. Ultrasound examination was suggestive of fatty liver and chronic kidney disease with cysts. Considering the characteristic manifestations of MYH9-RD described in the literature, a peripheral smear was performed, and typical inclusion bodies in the neutrophils were observed under a light microscope using Wright–Giemsa staining (Figure 1A). Ophthalmic examination revealed binocular high myopia, while the refractive stroma of both eyes was transparent, indicating that the patient did not have typical MYH9-related ophthalmopathy, i.e., cataract. Renal puncture could not be performed because of the consistently low levels of blood platelets. Other medical history included hypertension for 6 years and diabetes mellitus for 1 week. Medications included levamlodipin beslate (2.5 mg) qd and repaglinide (0.5 mg) tid to control blood pressure and glucose. Her family history included thrombocytopenia (her mother).

**Figure 2:**
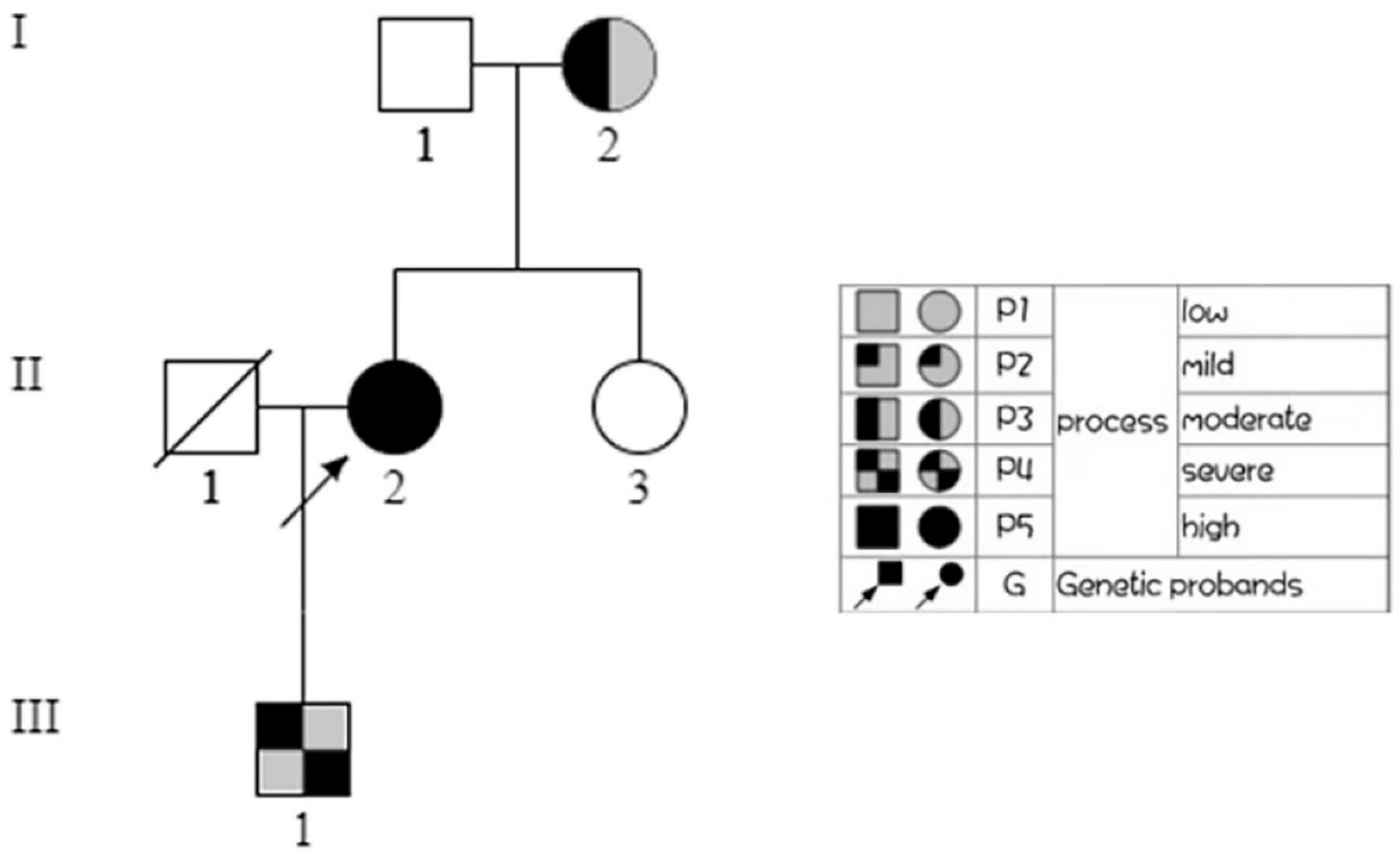
Family tree. In the family tree of this pedigree, the pattern differs as the severity differs. The severity is roughly dependent on the number of MYH9-RD related manifestations. The white pattern indicates physiological condition, while the all black pattern indicates the most severe.

To make a precise diagnosis, we performed a gene test for MYH9-RD related genes. Sequencing analysis revealed a heterozygous mutation c.5797delC (p. R1933Efs^*^15) in both the patient and her son in the *MYH9* gene, but no mutations were found in the sister of the patient, who had normal manifestations (Figure 1B). The 23-year-old son of the patient, who appeared healthy, was found to have a decreased blood platelet count (62 × 10^9^/L) and increased levels of liver enzymes (ALT 127 U/L, AST 54 U/L). Regarding renal function, the serum creatinine (67 μmol/L) and the GFR (GFR-cr 128 ml/min) were within normal range, but the urine protein fluctuated between “2+” and “3+,” but his hearing was not impaired. When tracing the history of the family, the mother of the proband, who was 71 years old, seemed to have MYH9-RD because she had thrombocytopenia (29 × 10^9^/L) and severe deafness, which was later confirmed by whole-exome sequencing. After the clinical data of the pedigree members were collected, we noticed that the proband and her son shared similar disease spectrums related to MYH9-RD, with the exception of hearing loss, while her mother displayed a milder phenotype (Table 1). The family tree was drawn according to the clinical manifestations and sequencing results (Figure 2).

**Table 1:** Clinical manifestations of the family.

	**Thrombocytopenia**	**Döhle-like bodies**	**SNHL**	**Cataract**	**Nephropathy**	**Elevated liver enzymes**
Mother	+	+	+	–	–	–
Patient	+	+	+	–	+	+
Son	+	+	–	–	+	+

*Only typical MYH9-RD clinical manifestations are taken into consideration here, and “+” means one has this feature. SNHL, sensorineural hearing loss*.

### Diagnosis and Treatment

Considering the physical and laboratory examinations mentioned above, our patient was diagnosed with chronic kidney disease stage IV, congenital thrombocytopenia (MYH9-related disease), hypertension, and diabetes.

After admission, we initiated symptomatic treatment, including thrombopoietic drugs, antihypertensive drugs, and antidiabetic agents. The patient was educated to avoid drugs that affect platelet function to prevent bleeding. Intervention measures were taken for the son of the patient because he also had renal involvement. He was discharged with angiotensin receptor blockers (ARBs) to control proteinuria and body weight.

### Clinical Follow-Up

At her latest evaluation, 6 months after discharge, the serum creatinine level was stable at approximately 200 μmol/L with a quantitative urine protein level of 1.5 g/day and blood pressure of 126/78 mmHg. The serum creatinine level of the son of the patient was in the normal range, as before (64 μmol/L), while his proteinuria decreased with a quantitative urine protein level of 0.5 g/day.

### Functional Changes Predictive of c.5797delC Mutations

**Figure 3:**
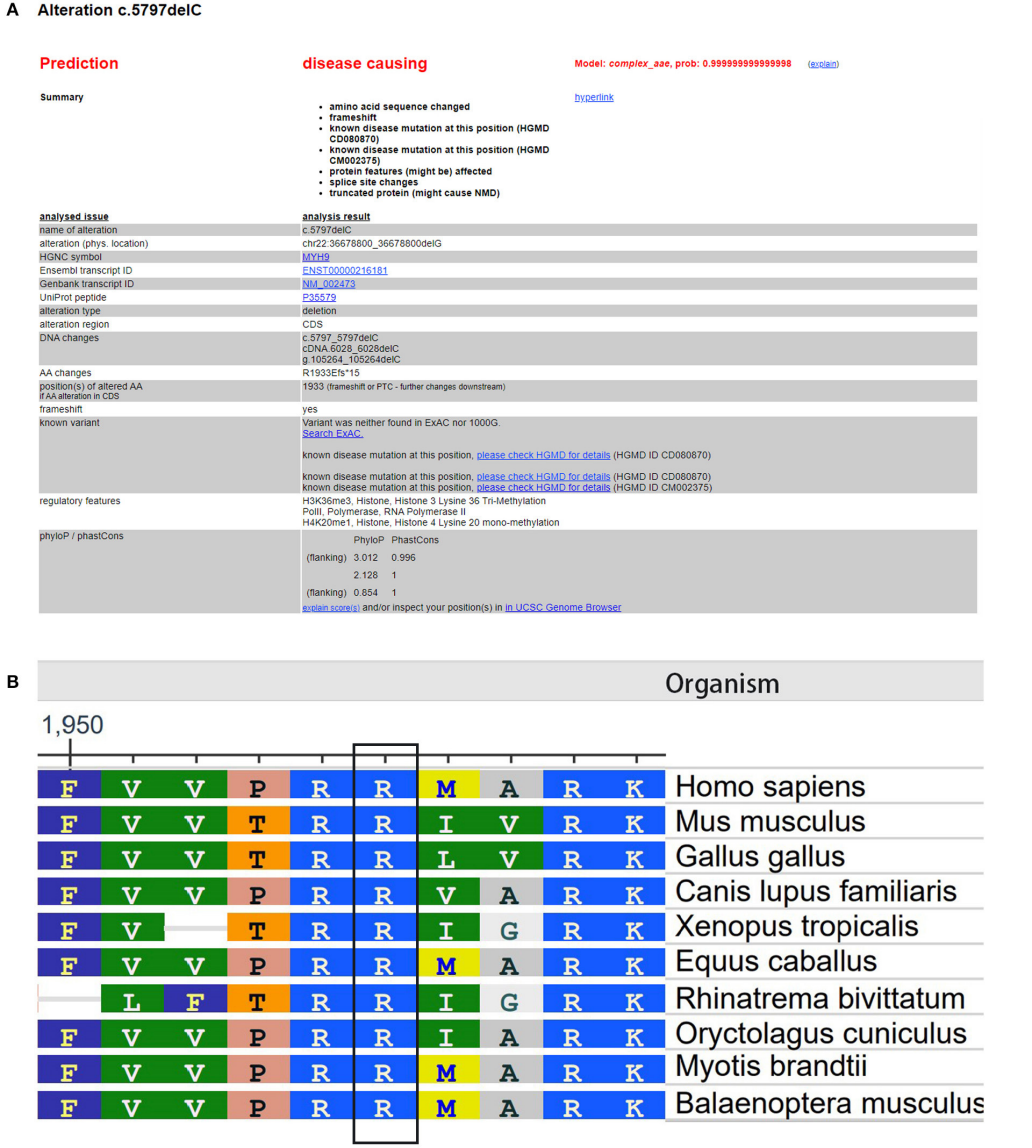
Evaluation of the pathogenic potential of the c.5797delC mutation in *MYH9*. **(A)** Web-based software Mutation Taster showed that this mutation was disease causing, and the mutation at this position was reported. **(B)** Analysis of amino acid conservation based on the NCBI database.

The Global Variome database showed that the c.5797delC mutation was pathogenic, but when we referred to the ClinVar database, we did not find a recorded item for this mutation. However, we found another substitution mutation at the same position, c.5795C>T, which was a known disease mutation in the ClinVar database. Web-based software Mutation Taster (http://www.mutationtaster.org/) predicted that this mutation was disease causing and capable of causing amino acid sequence changes, frameshift, and splice site changes (Figure 3A). In addition, arginine is an evolutionarily conserved amino acid in this protein among different organisms (Figure 3B), which indicates that changes in amino acids at this site may affect protein function.

## Discussion

In this study, a patient who presented with symptomatic thrombocytopenia was misdiagnosed with ITP and was later diagnosed with MYH9-RD. As it is a rare illness, other organ involvements such as the ear, eye, kidneys, and liver were not recognized initially; thus, making an accurate diagnosis was quite challenging. However, subsequent examinations revealed other involved organs, and the typical inclusion bodies in neutrophil granulocytes helped to confirm the diagnosis because neutrophil inclusions of myosin-9 are thought to be a pathognomonic sign of the disorder (Savoia et al., 2010). The sequencing results verified our hypothesis. Other affected individuals were found in this pedigree, and they all displayed clinical heterogeneity: menorrhagia as the initial symptom, with ear, kidney, and liver involvement in the patient; a milder phenotype with thrombocytopenia and deafness only in the mother; and proteinuria in the son. We also noticed that in this case, none of the three patients have eye involvement. We believe that the patient and her son share the same phenotype, but he may be in an early stage, since sensorineural hearing loss is present in approximately 50% of persons at the age of approximately 33 years (Pecci et al., 2014), and progressive development to chronic renal failure can be expected if no interventions are undertaken.

Here, the most obvious symptoms of the patient were thrombocytopenia and nephropathy. The mechanism of thrombocytopenia has been demonstrated to be due to MYH9-RD mutations impairing MK chemotaxis to disrupt migration toward the vasculature, thus, impairing pro-platelet release, and causing macrothrombocytopenia (Pal et al., 2020). However, the concrete mechanism of kidney involvement remains undefined. Generally, in the kidney, non-muscle myosin heavy chain IIA is expressed in podocytes; focal and segmental effacement of podocytes and loss of the interpodocyte slit diaphragm were observed under electron microscopy in MYH9-RD patients who presented with proteinuria and renal failure (Arrondel et al., 2002; Ghiggeri et al., 2003). Moreover, the *MYH9* gene is regarded as a risk factor for focal segmental glomerulosclerosis (FSGS), HIV-associated FSGS, hypertensive kidney failure, and non-diabetic ESRD in African Americans (Kao et al., 2008; Kopp et al., 2008). However, research based on a large Chinese IgA nephropathy cohort did not find any single-nucleotide polymorphisms that were associated with susceptibility to IgAN (Cheng et al., 2011). These studies showed that *MYH9* is related to kidney disease, but the association may vary among different ethnic groups.

What is unique about our case is that a patient with an NHT mutation, which is typically associated with a very “gentle” phenotype, as in the mother of the patient, developed a severe phenotype. We suggest that there may be other genes and/or environmental factors interacting with *MYH9* that induced this phenotype in the proband and her son because the *MYH9* mutation alone is not very convincing. Regrettably, whole-exome sequencing of this family did not reveal any notable findings. An *in vivo* study of mice with E1841K mutations in *MYH*9 indicated that the development of albuminuria or glomerular injury requires a second stimulus such as hypertension or loss of functioning nephrons (Cechova et al., 2018), which may partly explain why our patient had greater renal involvement. Her son presented with proteinuria only with normal renal function, and ARBs were administered to control the protein level, as these have been confirmed to be effective in reducing proteinuria in patients with MYH9-RD (Pecci et al., 2008a); this may prevent further renal involvement.

In conclusion, we present a case of a c.5797delC mutation in the *MYH9* gene in a 46-year-old Chinese woman with thrombocytopenia, inclusion bodies in neutrophils, sensorineural hearing loss, nephropathy, and abnormal liver enzymes, which are typical findings of MYH9-RD. Not all patients develop kidney disease with mutations; in particular, the chance of a mutation affecting the NHT domain is lower, so there must be some factors that we ignored. We would like to confirm this by performing genomic combined with metabolomic and proteomic testing to search for possible pathways that may interact with MYH9 and cause kidney disease in patients with MYH9-RD.

## Data Availability Statement

The original contributions presented in the study are included in the article/supplementary material, further inquiries can be directed to the corresponding author/s.

## Ethics Statement

Written informed consent was obtained from the patient for publication of this study and any accompanying images.

## Author Contributions

PR and HC participated in the experiments and wrote this article. YW, CW, and SF were responsible for the sample and information collection. HJ and JC guided the entire essay. All authors contributed to the article and approved the submitted version.

## Conflict of Interest

The authors declare that the research was conducted in the absence of any commercial or financial relationships that could be construed as a potential conflict of interest.

## Publisher's Note

All claims expressed in this article are solely those of the authors and do not necessarily represent those of their affiliated organizations, or those of the publisher, the editors and the reviewers. Any product that may be evaluated in this article, or claim that may be made by its manufacturer, is not guaranteed or endorsed by the publisher.
